# To Evaluate Different Endodontic Instrumentation Systems Regarding Post-Operative Pain After Endodontic Therapy: A Clinical Study

**DOI:** 10.7759/cureus.56466

**Published:** 2024-03-19

**Authors:** Bharath Gowda Govindiah Chandra Mohan, Disha Shivakumar, Sravana Laxmi Penumaka, Shaik Althaf, Garima Garg, Savadamoorthi Kamatchi Subramani

**Affiliations:** 1 Conservative Dentistry and Endodontics, Vokkaligara Sangha Dental College & Hospital, Bengaluru, IND; 2 Conservative Dentistry and Endodontics, Government Dental College and Hospital, Vijayawada, IND; 3 Conservative Dentistry and Endodontics, Oxford Dental College, Bangalore, IND; 4 Conservative Dentistry and Endodontics, Government Dental College and Hospital, Nagpur, IND; 5 Conservative Dentistry and Endodontics, College of Dentistry, Jazan University, Jazan, SAU

**Keywords:** endodontic therapy, post operative pain, protaper gold and hyflex edm file systems, oneshape, reciproc

## Abstract

Background: Despite substantial breakthroughs in instrumentation systems and pharmaceutical interventions, pain following endodontic therapy remains a serious concern. The effect of the type of endodontic instrumentation system in post-operative pain after endodontic therapy has been a matter of debate.

Aim: To evaluate different endodontic instrumentation systems, namely Reciproc (GmbH, Munich), OneShape® (MicroMega, France), Protaper Gold (Dentsply Sirona, USA), and Hyflex® EDM (Coltène/Whaledent Inc., USA) file systems, regarding post-operative pain after endodontic therapy

Methods and materials: The endodontic department treated healthy patients aged 20 to 50 years who were experiencing symptoms of irreparable pulpitis in one or more maxillary molars or mandibular molars. Five hundred was the determined size of the sample. The study participants were divided into five categories, each comprising 100 participants. These categories were: Category 1: Reciproc instrumentation system. Category 2: OneShape® instrumentation system. Category 3: ProtaperGold instrumentation system. Category 4: HyFlex® EDM instrumentation system. Category 5: Control (stainless steel K-files). Following endodontic therapy, these scores were recorded at 6 hours, 12 hours, 24 hours, 48 hours, and 72 hours using the VAS scale.

Results: The visual analog scale (VAS) score (mean±SD) in the control group was 0.73± 0.40 (<0.001). The VAS score in the Reciproc group was 0.43± 0.05 (<0.001). The VAS score in the OneShape® group was 0.36±0.09 (<0.001). The VAS score in the Protaper Gold group was 0.41 ±0.08 (<0.001). The VAS score in the HyFlex® EDM group was 0.55 ±0.02 (<0.001). The VAS score in all instrumentation techniques at 72 hours follow-up was lesser in comparison to a control group with meaningful statistical significance (<0.001). However, the post-operative pain among the Reciproc, OneShape®, Protaper Gold, and HyFlex® EDM instrumentation systems was not different clinically when compared among themselves. However, VAS values were greater in OneShape® and HyFlex® EDM compared to Reciproc and Protaper Gold, showing increased post-operative pain in OneShape and HyFlex® EDM compared to Reciproc and Protaper Gold. It was also observed that there was a decline in the VAS score in all instrumentation systems as the follow-up period increased from 6 hours to 72 hours, with maximum post-operative pain at 6 hours of follow-up and minimum post-operative pain at 72 hours of follow-up. However, the decline was lesser in OneShape® and HyFlex® EDM in comparison to Reciproc and Protaper Gold, with increased post-operative pain in OneShape® and HyFlex® EDM in comparison to Reciproc and Protaper Gold.

Conclusion: Post-operative pain at all follow-ups of endodontic procedures was less in Reciproc, OneShape®, Protaper Gold, and HyFlex® EDM than in the control group. VAS scores were higher in the OneShape® and HyFlex® EDM groups compared to the Reciproc and Protaper Gold groups, indicating increased post-operative pain with OneShape® and HyFlex® EDM instruments in comparison to Reciproc and Protaper Gold.

## Introduction

With prevalence rates varying from 1.9 percent to 48%, according to published research, pain after completion of endodontic therapy continues to be a significant issue despite significant advancements in instrumentation systems and pharmaceutical therapies [1,2]. This wide range is most likely the result of variations in post-operative pain definitions and study designs [3,4]. Post-operative endodontic pain has been documented in the scientific literature, with both severe intensities observed in 6-12% of cases and mild intensity observed in 10 to 30 percent of cases, even in the presence of the strictest criteria [5,6].

Pain after endodontic therapy postoperatively can be caused by several etiologic variables, such as ejection of detritus into tissue in the periapical region, history of pre-operative pain, hyper occlusion, poor canal cleaning, and periapical illness [7,8]. One main cause of pain following orthodontic therapy has been proposed to be the protrusion of diseased dental material into the area around the root apex [9,10]. Regardless of the instruments being restricted to the root canal's boundaries, the process of detritus protrusion is an unavoidable phenomenon [11,12]. However, varying root canal instrumentation systems appear to be linked to varying degrees of extrusion [13-16]. Some research reveals higher levels of extruded contaminants following the use of hand endodontic files instead of engine-driven endodontic files [17-21].

Reciproc is an endodontic NiTi single-file apparatus that promises to accomplish both the shaping of the root canal as well as the cleaning of the root canal with just one file [10,11]. An electronic motor must be equipped with three separate files, namely R25 with a 25/0.08 dimension, R40 with a 40/0.06 dimension, and R50 with a 50/0.05 dimension, that utilize the reciprocating movement in this instrumentation system. The M-wire alloy used to make the files offers increased resilience to cycle fatigue and greater flexibility. The other endodontic single-file NiTi system, called OneShape®, comes in three different sizes, which are 25/0.06 size, 30/0.06 size, and 40/0.06 size, and it employs full-sequence rotating movement [11,12]. The file features a longer pitch and varying cross-sections. These characteristics result in a shorter root canal preparation time, effective root canal cleaning, a bacterial charge reduction comparable to that of a conventional instrument system, and a reduction in the amount of apically expelled debris [22-23]. It employs an orifice shaper (EndoFlare) to remove the occlusal restrictions and has flexible cross sections [24-26].

Numerous studies conducted over the past few decades have resulted in the introduction of rotary endodontic instrument systems with different tapers and increasingly flexible NiTi instruments called Protaper Gold [13,14]. The manufacturer claims that it is identical to Protaper Universal (Dentsply Sirona, USA) in geometry, with the sole difference being that it is more flexible than the former. Additionally, the manufacturers asserted that it could preserve the root canal centering ability and resist cycle fatigue. Electrical discharge machining, or EDM, is a recent and significant advancement in endodontic file manufacturing that has the advantage of elevating endodontics to a new level. Using an innovative method, electric discharge machining was used to create EDM endodontic files that are resistant to fracture and have more strength [13,15,17]. Because of this unique combination of flexibility and fracture resistance, fewer files are required to shape and clean the root canal without altering or harming the root canal's architecture.

Endodontic treatment can be provided in one or more visits; single visits have several benefits, such as fewer sittings required, no possibility of leakage between appointments, shorter treatment times overall, and low cost [27-30]. Achieving minimum intrusive preparations while preserving as much tissue as possible is the ultimate goal for endodontically treated dental restorations [31-35]. Stainless steel K-files are essential instruments in endodontic therapy, offering durability, flexibility, and precision for the cleaning and shaping of root canals. Their straightforward design, wide range of sizes, and reliable performance make them indispensable tools for root canal treatment procedures [36].

The role of the type of endodontic instrumentation system in post-operative pain after endodontic therapy has been a matter of debate [36-39]. To the best of the author's knowledge, no study in the past has evaluated different endodontic instrumentation systems, namely Reciproc (GmbH, Munich), OneShape® (MicroMega, France), Protaper Gold (Dentsply Sirona, USA), and Hyflex® EDM (Coltène/Whaledent Inc., USA) file systems, regarding postoperative pain after endodontic therapy. Therefore, this study was carried out to compare post-operative pain after endodontic therapy with these different endodontic instrumentation systems. 

## Materials and methods

Study design

This study was designed as a clinical study to evaluate the effectiveness of different endodontic instrumentation systems in treating irreparable pulpitis. 

Participants

Participants were recruited from the endodontic department of a dental college and hospital. Inclusion criteria included healthy patients aged 20 to 50 experiencing symptoms of irreparable pulpitis in one or more maxillary or mandibular molars. Exclusion criteria comprised individuals who had previously received endodontic therapy, recent medication intake, pregnancy, complicated root canal anatomy, root canal calcifications, tooth resorption, periodontal disorders, swelling or abscess, pathology around the root apex, sensitivity to percussion, and absence of occlusal contact. Each participant provided informed consent and relevant medical history before enrollment.

Sample size determination

Based on previous studies and assuming a significance level (α) of 0.05 and a power (β) of 80%, a sample size of 500 participants was determined. Participants were divided into five categories, each comprising 100 individuals.

Randomization and blinding

Participants were randomly assigned to treatment groups using a random number generator. Allocation concealment was ensured by sealing treatment assignments in opaque envelopes, which were opened by the treating endodontist only after determining the functional root canal length. Both participants and practitioners were blinded to treatment assignments to minimize bias.

Interventions

The five treatment categories included were Group 1 as the Reciproc instrumentation system, Group 2 as the OneShape® instrumentation system, Group 3 as the ProtaperGold instrumentation system, Group 4 as the HyFlex® EDM instrumentation system, and Group 5 as the control group (stainless steel K files). 

Treatment procedures followed manufacturer-recommended protocols for each instrumentation system. Anesthesia was administered using 2% lidocaine with 1:80000 epinephrine, and rubber dams were used for isolation. Access cavities were prepared, and root canals were instrumented accordingly. Irrigation and obturation protocols were standardized across all groups.

Outcome measures

The primary outcome was post-operative pain intensity assessed using the visual analog scale (VAS) at 6, 12, 24, 48, and 72 hours after treatment. Secondary outcomes included working time and adverse events. After drying the root canals with paper cones, they were sealed with AH-26 sealer and gutta-percha. After that, Cavit was used to temporarily seal the tooth. For pain management, the patients were instructed to take 400 mg of Ibuprofen as needed [24,28]. Patients were given VAS questionnaires after the session and asked to indicate on the form what number best reflected their level of pain following treatment (0 being no pain and 10 being the highest level of agony possible). Following endodontic therapy, these scores were recorded by two operators (both dentists in the post-graduate program of dentistry/endodontics) at durations of 6 hours, 12 hours, 24 hours, 48 hours, and 72 hours. Unaware of the intervention groups, a different analyzer called study participants at the scheduled follow-ups and gathered the VAS scores.

Statistical analysis

Data were analyzed using IBM SPSS Statistics V21.0. Non-parametric tests, including Mann-Whitney U and Kruskal-Wallis tests, were used due to the non-normal distribution of the data. Statistical significance was set at p < 0.05.

Ethical considerations

The ethical committee of Vokkaligara Sangha Dental College and Hospital approved the study protocol with institutional review board (IRB) number IEC/VSDC&H/2021/27. Informed consent was obtained from all participants, and the study was conducted following ethical principles outlined in the Declaration of Helsinki.

## Results

Demographic details of study participants are shown (Table 1).

**Table 1:** Demographic details of study participants EDM: Electrical discharge machining; SD: Standard deviation

Demographic and clinical data	Male	Female	Mean age ± SD (years)	Maxillary molar	Mandibular molar
Control	46	54	32.8 ±5.93	50	50
Reciproc	38	62	34.3 ±5.41	42	58
OneShape	42	58	31.3 ±4.39	40	60
Protaper Gold	39	61	33.4 ±4.38	45	55
Hyflex EDM file	43	57	34.3±4.32	43	57

The control group had 46 male and 54 female participants, with a mean age of 32.8 ± 5.93 years. The group consisted of 50 maxillary molars and 50 mandibular molars. In the Reciproc group, there were 38 male and 62 female participants, with a mean age of 34.3 ± 5.41 years. The group included 42 maxillary molars and 58 mandibular molars. The OneShape® group had 42 male and 58 female participants, with a mean age of 31.3 ± 4.39 years. This group comprised 40 maxillary molars and 60 mandibular molars. The ProTaper Gold group consisted of 39 male and 61 female participants, with a mean age of 33.4 ± 4.38 years. There were 45 maxillary molars and 55 mandibular molars in this group. Finally, the HyFlex® EDM file group included 43 male and 57 female participants, with a mean age of 34.3 ± 4.32 years. This group comprised 43 maxillary molars and 57 mandibular molars. VAS scores in different instrumentation systems at different follow-up post-operative endodontic therapy are shown (Table 2).

**Table 2:** VAS score in different instrumentation systems at different follow-up post-operative endodontic therapy EDM: Electrical discharge machining; SD: Standard deviation; VAS: Visual analog scale

Variables	VAS score (Mean±SD)	VAS score (Mean±SD)	VAS score (Mean±SD)	VAS score (Mean±SD)	VAS score (Mean±SD)	VAS score (Mean±SD)	p-value
Groups	Baseline	6 hrs follow-up	12 hrs follow-up	24 hrs follow-up	48 hrs follow-up	72 hours follow-up	
Control	7.47±3.17	5.23±2.81	3.99 ±2.71	2.87 ±0.31	1.63±0.01	0.73± 0.40	<0.001
Reciproc	7.71±2.33	3.34±3.25	1.63±2.64	0.89±0.01	0.52±0.06	0.43± 0.05	<0.001
OneShape	6.43±2.81	3.62±3.03	1.81 ±2.28	0.86±0.52	0.67±0.07	0.36±0.09	<0.001
Protaper Gold	7.54±2.36	3.13±3.25	1.72±2.41	0.91±0.03	0.54±0.03	0.41 ±0.08	<0.001
Hyflex EDM file	6.26±2.93	3.26±2.02	1.81±2.29	0.92±0.43	0.61±0.02	0.55 ±0.02	<0.001
p-value	0.074	0.006	< 0.001	< 0.001	< 0.001	< 0.001	<0.001

For the control group, the VAS scores decreased progressively from 7.47 ± 3.17 at baseline to 0.73 ± 0.40 at 72 hrs follow-up, with statistically significant differences observed at all time points (p < 0.001). Similarly, in the Reciproc group, VAS scores decreased significantly from 7.71 ± 2.33 at baseline to 0.43 ± 0.05 at 72 hrs follow-up (p < 0.001). In the OneShape® group, VAS scores decreased significantly from 6.43 ± 2.81 at baseline to 0.36 ± 0.09 at 72 hrs follow-up (p < 0.001). For the ProTaper Gold group, VAS scores decreased significantly from 7.54 ± 2.36 at baseline to 0.41 ± 0.08 at 72 hrs follow-up (p < 0.001). Similarly, in the HyFlex® EDM file group, VAS scores decreased significantly from 6.26 ± 2.93 at baseline to 0.55 ± 0.02 at 72 hrs follow-up (p < 0.001).

## Discussion

The role of the type of endodontic instrumentation system in post-operative pain after endodontic therapy has been a matter of debate. According to Bürklein et al. [10], Reciproc, a single endodontic file reciprocating instrumentation system, generated more debris outflow than OneShape® as well as F360, two single file endodontic rotary systems. 

Several etiologic factors can contribute to pain after endodontic therapy postoperatively, including detritus ejection into the periapical tissue, pre-operative pain history, hyper occlusion, inadequate canal cleaning, and periapical disease [25,26]. It has been suggested that the protrusion of defective denture material into the area surrounding the root apex is one of the primary causes of pain after orthodontic therapy [27,28]. Debris expulsion into the periapical area is known to cause irritation in tissues in areas around the root apex and induce inflammation, which can result in outbreaks and discomfort following endodontic therapy [5,30]. Although some research has found that reciprocating rotary instrumentation systems have less debris expulsion than full-sequence rotary endodontic instrumentation systems [7,30], other investigations have found that reciprocating rotary instrumentation systems demonstrate greater debris expulsion [8,9,32]. Differences in the cutting efficacy, kinematics, number of used files, cross-section, configuration, tape, cutting-edge design, and other factors could be the cause of the variation that was observed [32-38].

Despite substantial breakthroughs in instrumentation systems and pharmaceutical interventions [32,34], pain following endodontic therapy completion remains a serious concern, with incidence rates ranging from 1.9 percent to 48%, according to published research [31,33]. Variations in post-operative pain classifications and study designs are most likely the cause of this significant heterogeneity [33,35]. Even in the face of the toughest criteria, post-operative endodontic pain has been reported in the scientific literature as mild in ten to thirty percent of cases and severe in six to twelve percent of cases [30,34].

This study also observed a decline in VAS score in all instrumentation systems as the follow-up period increased from 6 hours to 72 hours, with maximum post-operative pain at 6 hours follow-up and minimum post-operative pain at 72 hours follow-up. However, the decline was lesser in OneShape® and HyFlex® EDM in comparison to Reciproc and Protaper Gold, with increased post-operative pain in OneShape® and HyFlex® EDM. Neelakantan et al. [18] found that patients receiving root canal biomechanical preparation with Reciproc compared to OneShape experienced considerably less duration and severity of post-operative discomfort. This was based on randomized multicenter clinical research.

In 2011, Pak and White conducted a systematic analysis and found that the early stages of randomized controlled trials had the greatest incidence of post-operative discomfort [37]. Post-operative pain occurred 40% of the time in the first 24 hours, then declined over the next 48 hours to 11% or lower on the seventh day. The motion kinematics of the instrumentation system are the most crucial thing to consider. There was reduced post-operative endodontic pain when utilizing a rotational file system instead of a reciprocal single file system. The ejection of debris was the reason for the variation across the two categories, based on the instrumentation approach used [38,39]. Several in vitro experiments comparing reciprocal instrumentation systems and rotary instrumentation systems concluded that the application of endodontic root canal preparation instruments in reverse movement caused the greatest amount of debris ejection [35,36].

The study has some limitations, as the sample size of 500 participants may seem substantial, but larger cohorts could offer more reliable and generalizable insights. With a larger sample size, the study would have greater statistical power, allowing for more precise estimates of the true effects. This is particularly important in healthcare research, where variability among individuals can significantly influence outcomes. Additionally, larger sample sizes can help detect smaller yet clinically significant differences between groups or treatments. The study's findings may have limited generalizability due to specific inclusion criteria. The study may not reflect the broader population of patients undergoing endodontic therapy by restricting participants to healthy individuals aged 20 to 50 with irreparable pulpitis in specific molar teeth. Patients with different demographic characteristics, medical histories, or types of dental conditions may respond differently to treatment and experience varying levels of post-operative pain. Therefore, the results may not apply to a more diverse patient population in clinical practice. Relying solely on the VAS to assess post-operative pain may not capture the full spectrum of patient experiences. While the VAS is a commonly used and validated tool for pain assessment, it primarily measures pain intensity at a specific point in time. However, pain experiences are multifaceted and can encompass various dimensions, such as duration, quality, and impact on daily activities. Incorporating additional outcome measures, such as pain duration, pain interference with daily functioning, or patient-reported satisfaction with treatment, could provide a more comprehensive understanding of post-operative pain outcomes. Although the study recorded pain scores at multiple follow-up intervals up to 72 hours post-treatment, longer-term follow-up assessments were not conducted. Extended follow-up periods would offer valuable insights into the sustained effectiveness of treatment and the resolution of post-operative pain over time. Some patients may experience fluctuations in pain intensity beyond the initial post-operative period, and long-term follow-up assessments would capture these dynamics. Understanding the durability of pain relief and treatment outcomes is essential for guiding clinical decision-making and optimizing patient care.

## Conclusions

Post-operative pain at all follow-ups of endodontic procedures was less in Reciproc, OneShape®, Protaper Gold, and HyFlex® EDM as compared to the control. However, the post-operative pain among the Reciproc, OneShape®, Protaper Gold, and HyFlex® EDM instrumentation systems was not different clinically when compared among themselves. However, VAS values were greater in OneShape® and HyFlex® EDM in comparison to Reciproc and Protaper Gold, showing increased post-operative pain in OneShape® and HyFlex® EDM in comparison to Reciproc and Protaper Gold. These results underscore the importance of selecting appropriate instrumentation systems in endodontic therapy to optimize patient comfort and treatment outcomes. However, further research with larger sample sizes, randomized controlled designs, and longer-term follow-up is warranted to validate these findings and elucidate the factors contributing to post-operative pain variability among different instrumentation systems.
